# Results of 198 primary total hip arthroplasties using the Delta PF-FIT system with ceramic-on-ceramic articulating surfaces with average seven years follow up

**DOI:** 10.1186/s12891-020-03253-x

**Published:** 2020-05-19

**Authors:** Petr Fulin, David Pokorny, Jan Hert, Antonin Sosna

**Affiliations:** Orthopaedic Clinic 1st Faculty of Medicine Charles University and Motol University Hospital, V Uvalu 84, 15006 Prague, Czech Republic

**Keywords:** Total hip arthroplasty, Delta PF cup, FIT stem, LimaCorporate, HHS, WOMAC

## Abstract

**Background:**

The lifetime implants is a key parameter that the surgeon should take into account at the time of the primary total hip arthroplasty (THA). The aim of this study was a clinical and radiographical evaluation of the Delta PF-FIT (LimaCorporate, Italy) THA system with ceramic-on-ceramic articulations. We have not found a clinical or radiographical assessment of this implant in available published literature.

**Methods:**

A total of 197 (F = 94, M = 103) primary THAs were evaluated in 163 patients with a mean follow-up of 7.7 years (range 5.1–11.2 years (SD ± 1.5)) Harris hip Score (HHS) and the Western Ontario and McMaster Universities Arthritis index (WOMAC) were used for the clinical evaluation. The statistical evaluation was processed by standard statistical methods. The study was approved by Ethic Committee of the University Hospital Motol (Reference No. EK-73/19).

**Results:**

The mean HHS score was found to be 97.59 points (61–100 range with a ± 5.13 SD, preoperative HSS was 51.21, range 28–73 with a ± 4,77 SD). 186 THAs were evaluated as excellent (90–100 points), 9 THAs rated as good (80–89 points), 1 THA was rated as fair (70–79) points and 1 THA rated as poor (less than 70 points). The mean WOMAC score was 97.38 points (65–100 range with a ± 5.18 SD, preoperative was 50,12, range 27–69 with a ± 4.85 SD). We documented an overall 99.49% Kaplan-Meier survival with a mean follow-up of 7.7 years with the FIT (LimaCorporate) stem revision and any component revision as the endpoint. With the Delta PF (LimaCorporate) cup revision as the endpoint, the survival was 100%. We have not found a previously published clinical or radiographical review of this THA system, the study shows a comparison with other THA implants.

**Conclusion:**

Evaluation of the Delta-PF-FIT (LimaCorporate, Italy) THA system with the use of ceramic-on-ceramic BIOLOX®Delta articulation surfaces shows very good outcomes.

## Background

Total hip replacement is the current proven mothod of treating many ailments of the hip joint. Worldwide, there is an increasing number of primary Total Hip Arthroplasties (THAs), along with THA revision surgeries as a consequence. As the indications for THA widen, patients that undergo surgery are more active and have longer lifespans. The lifetime of the implants is therefore a key parameter that the surgeon should take into account at the time of the primary THA. The surgeon should consider the expected level of activity of the patient; their biological and chronological age and associated diseases. The choice of articulating surfaces is a major factor in the expected lifetime of the implant. According to studies by Amanatullah, the ceramic-on-ceramic pairing of articulating surfaces offers the best friction properties [1]. The aim of this study is a clinico-radiographical assessment of the Delta PF-FIT (LimaCorporate, Italy) THA system with a ceramic-on-ceramic paring of articulating surfaces.

## Methods

**Fig. 1:**
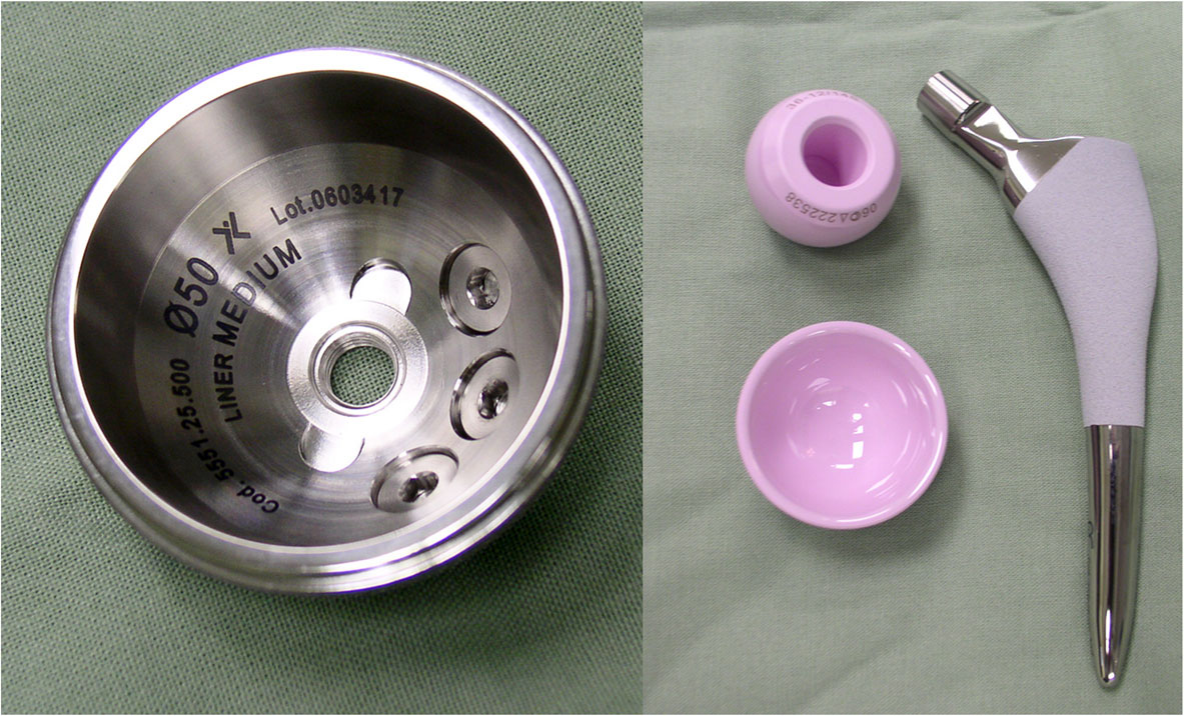
Lima Implants (LimaCorporate). Delta PF Cup (left), FIT stem with ceramic insert and a BIOLOX® Delta head (right)

The Delta PF is a hemispherical cementless acetabular cup manufactured from Titanium alloy (Ti6Al4V), available in Italy from 2003 and in the Czech Republic from 2006. The surface finish of the cup has a coating of porous titanium and an additional layer of hydroxyapatite in order to stimulate bone growth and osseointegration of the cup [2]. The cup has equatorial retentive grooves for optimal press-fit effect (Fig. 1). The acetabular cup is available in sizes ranging from 44mm to 66mm in 2mm increments. The inserts for the Delta PF cup have a patented peg at the rear for precise insertion into the metal cup. This prevents malposition of the insert, which may lead to splintering of the edge of the insert and possible early failure [2]. It also acts as a prevention for the concentration of forces in the ceramic material. The FIT stem is a titanium (Ti6Al4V), cementless anatomical prosthesis that has been on the market in the Czech Republic since 2005. The proximal part of the stem has porous titanium with a coating of hydroxyapatite for optimal implant integration. The stem was designed to offer maximum stability while respecting the morphology of the proximal femur (Fig. 1). The result of which is a physiological force transfer in the region of proximal femur similar to that of a healthy femur. The medial anatomical curve of the stem balances out the 9° lateral curve in the base of the stem isthmus. Due to the asymmetrical shape of the implant, it ideally fills out the femoral canal and prevents abnormal force transfer to surrounding cortical bone; reducing the risk of stress shielding and thigh pain [2].

**Fig. 2:**
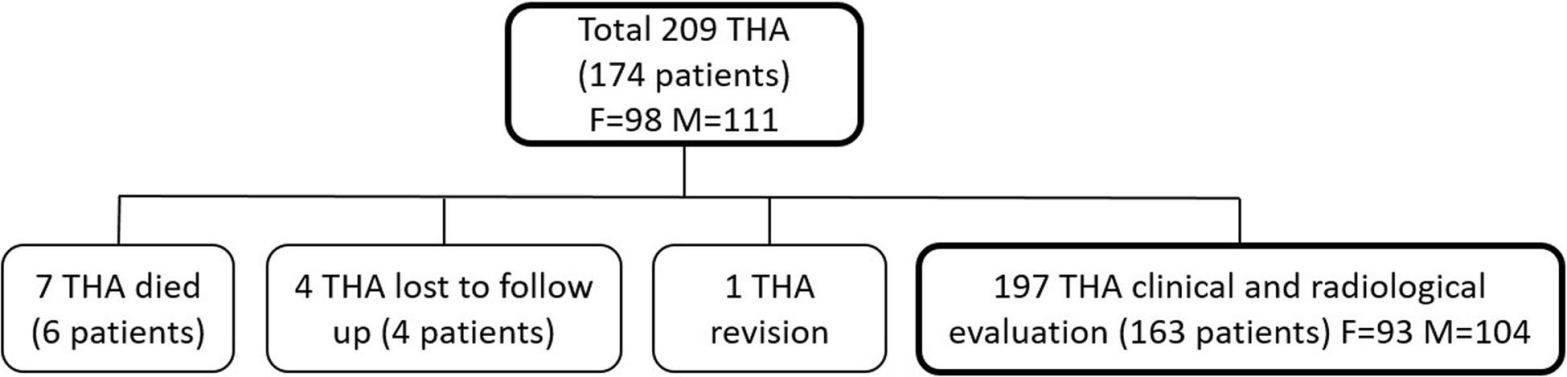
Distribution of operated patients

Between 2006 and 2012, we implanted 209 Delta PF-FIT (LimaCorporate, Itálie) THAs with ceramic-on-ceramic (BIOLOX®Delta) articulation surfaces in 174 patients at our clinic. Six patients (7 hips) died within the follow-up period and 4 patients (4 hips) were lost to follow-up. In one particular case, a revision was performed due to a periprosthetic fracture of the femur. Overall, 197 THAs were assessed both radiographically and clinically. (F = 94, M = 103) in 163 patients (Fig. 2). The mean age at time of surgery was 57.8 years (range 24 - 81 years SD ± 10,6). The left hip was operated in 103 cases and the right in 94 cases. The mean time of follow-up was 7.7 years (range 5.1 - 11.2 years SD ± 1,5). The indications for THA are summarised in a table (Table 1).

**Table 1:** Indications for THA

Indications		
Diagnosis	Number	Percentage
Arthritis	153	77.7%
Postdysplastic arthritis	23	11.7%
Femoral head necrosis	10	5.1%
Rheumatic arthritis	4	2.0%
Femoral neck fracture	4	2.0%
Femoral nail failure	2	1.0%
State after coxitis	1	0.5%

Clinical evaluation was done with the use of the Harris Hip Score (HHS) [3] and Western Ontario and McMaster Universities Arthritis index (WOMAC Score) [4] pre- and post-operatively. The HHS evaluates the clinical and the functional result. The result is rated based on a set grading: Excellent (90-100 points), Good (80-89 points), fair (70-79 points) and poor (less than 70 points). The WOMAC score evaluates pain, rigidity, other symptoms and function in daily life. (0-100 scale). The radiographical assessment evaluated the zone of translucency surrounding the femoral and acetabular components, cortical hypertrophy of the femur surrounding the stem, subsidence of the stem when compared to the postoperative radiograph, changes in cup orientation and the occurrence of periarticular ossifications.

All THAs were performed by 3 very experienced surgeons. In all cases the surgical approach was anterolateral with the patient in supine position. The cup was implanted by press-fit method in an inclination angle of 45° and anteversion angle of 10-15°, a ceramic Biolox®Delta insert was then inserted into the cup. The stem was implanted anatomically with the press-fit method. A Biolox®Delta ceramic head was used in all cases.

The statistical evaluation included the calculation of mean, median andinterquartile range (IQR) values for selected variables. To compare the different hip replacement systems in the discussion, we also set up Kaplan-Meier survival analysis with 95% confidence intervals by using the Statistica 12 program (StatSoft, USA). We used scatterplot matrix graph and Pearson’s and Spearmen’s correlation coefficients for more detailed correlations of individual measurement values.

The study was approved by Ethic Committee of the University Hospital Motol (Reference No. EK-73/19). This is a retrospective study.

## Results

Overall, 198 THAs out of the original 209 were evaluated (7 died, 4 lost to follow-up). One patient was revised due to a periprosthetic fracture of the femur (revision rate 0,51%). We had no infectious complications. Therefore, 197 hips were assessed both clinically and radiographically.

### Clinical outcome assessment

In 2 patients there were intraoperative complications; those being the formation of a fissure in the proximal femur during the impaction of the femoral stem, without affecting the primary stability of the implant. In both cases, spontaneous healing of the fracture occurred with a good final function of the implanted hip. Among post-operative complications, the most common was a limitation in the range of movement. A hip flexion contracture of up to 10° occurred in 4 cases. A slight, intermittent pain was reported by 2 patients; one patient reported a slight pressure in the thigh during heavy loads. The average range of flexion was 104.16° (70 – 130° range with a 10,0 IQR). The average range of abduction was 40.72° (30 – 50° range with a 5,0 IQR). The average range of external rotation was 28.95° (0 – 40° range with a 0,0 IQR). The average range of internal rotation was 19,13° (0 – 35° range with 2,0 IQR) (Table 2). The mean HHS score was 97.59 points (61-100 range with a 3,0 IQR). 186 THAs were evaluated as excellent (90-100 points), 9 THAs rated as good (80-89 points), 1 THA was rated as fair (70-79 points) and 1 THA rated as poor (less than 70 points). Pre-operatively mean HSS was 51.21 (Table 3) and HHS increased in average by 46.38 points. The post-operatively mean WOMAC score was 97.38 points (65-100 range with a 4,0 IQR). Pre-operatively mean WOMAC was 50.12 (Table 3) and WOMAC increased in average by 47.26 points. In both cases, the rise in scores is statistically significant (p ≤ 0,05).

**Table 2:** Statistical values (FU-follow up, FL- flexion, ABD – abduction, ER – external rotation, IR- internal rotation, IQR interquartile range)

	Age	FU	HHS	WOMAC	FL	ABD	ER	IR
mean	57.9	7.7	97.6	97.4	104.2	40.7	28.9	19.1
median	59.0	7.7	100.0	100.0	100.0	40.0	30.0	20.0
*5% trimmed mean*	*58.5*	*7.6*	*98.3*	*98.2*	*104.3*	*40.9*	*29.1*	*19.3*
IQR	11.0	1.9	3.0	4.0	10.0	5.0	0.0	2.0

**Table 3:** Results of pre- and post-operatively clinical evaluation (SD – standard deviation)

Results (*n* = 197)								
	Preoperative				Postoperative			
	Mean	Min	Max	SD	Mean	Min	Max	SD
HSS	51.21	28	73	4.92	97.59	61	100	5.13
WOMAC	50.12	27	69	4.94	97.38	65	100	5.18
Flexion	89.51	45	110	8.2	104.16	70	130	10.61
Abduction	27.32	5	40	4.71	40.72	30	50	4.52
External rotation	19.81	0	30	5.12	28.95	0	40	5.6
Internal rotation	11.26	0	20	4.84	19.13	0	35	6.17

### Radiographic evaluation

**Fig. 3:**
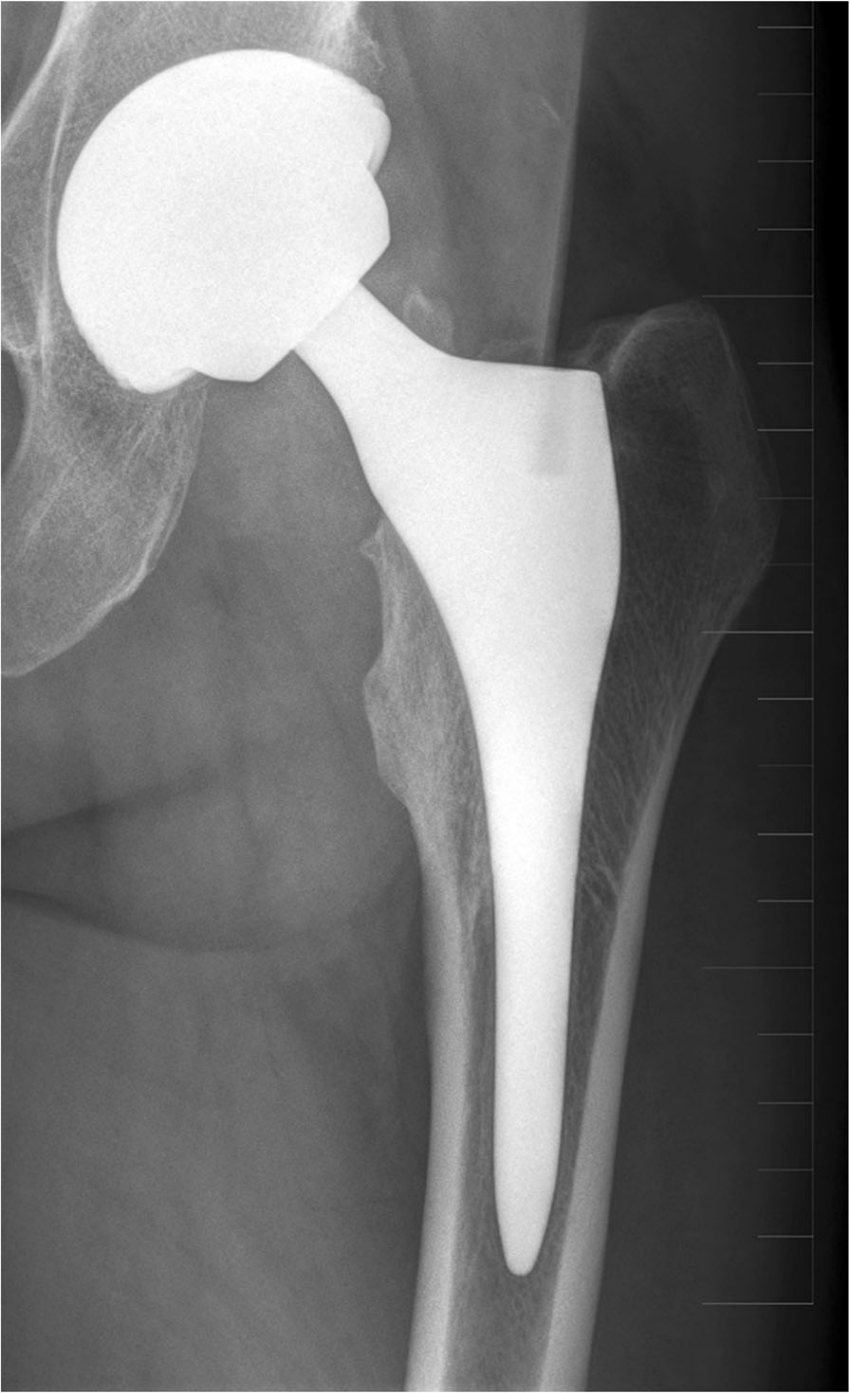
Bone trabeculae remodelling with the integration into the hydroxyapatite surface of the implant

Radiolucent lines surrounding the acetabular or femoral components have not been observed in a single case in our cohort. There were also no changes in orientation or change of position of the acetabular cup. A slight femoral bone hypertrophy surrounding the stem (spot weld) was observed in one case. In two patients, a slight stem subsidence (approx. 5mm) of the femoral component was observed when compared to the postoperative radiograph. Both patients however have no functional limitation and are without problems. In both cases, the cause was most likely an incorrect selection of an undersized implant. A further 15 hips were noted as having an undrsized implant. Despite this, there has not been a single case of loosening; all patients are without problems. In these hips, we observed the remodelling of bone trabeculae with their integration onto the surface of the prosthesis (Fig. 3). In 11 patients, we observed a slight varus orientation of the stem on an antero-posterior radiograph without any corelating clinical problems. Wear of articulating surfaces was not been observed in a single case.

The occurrence of heterotopic ossifications was evaluated according to Brooker’s classification [5]. Grade I was identified in 25 cases (12,7%), grade II in 16 cases (8,1%) and grade III in 5 cases (2,5%). We did not observe any cases with Grade IV ossifications.

### Statistical analysis

**Fig. 4:**
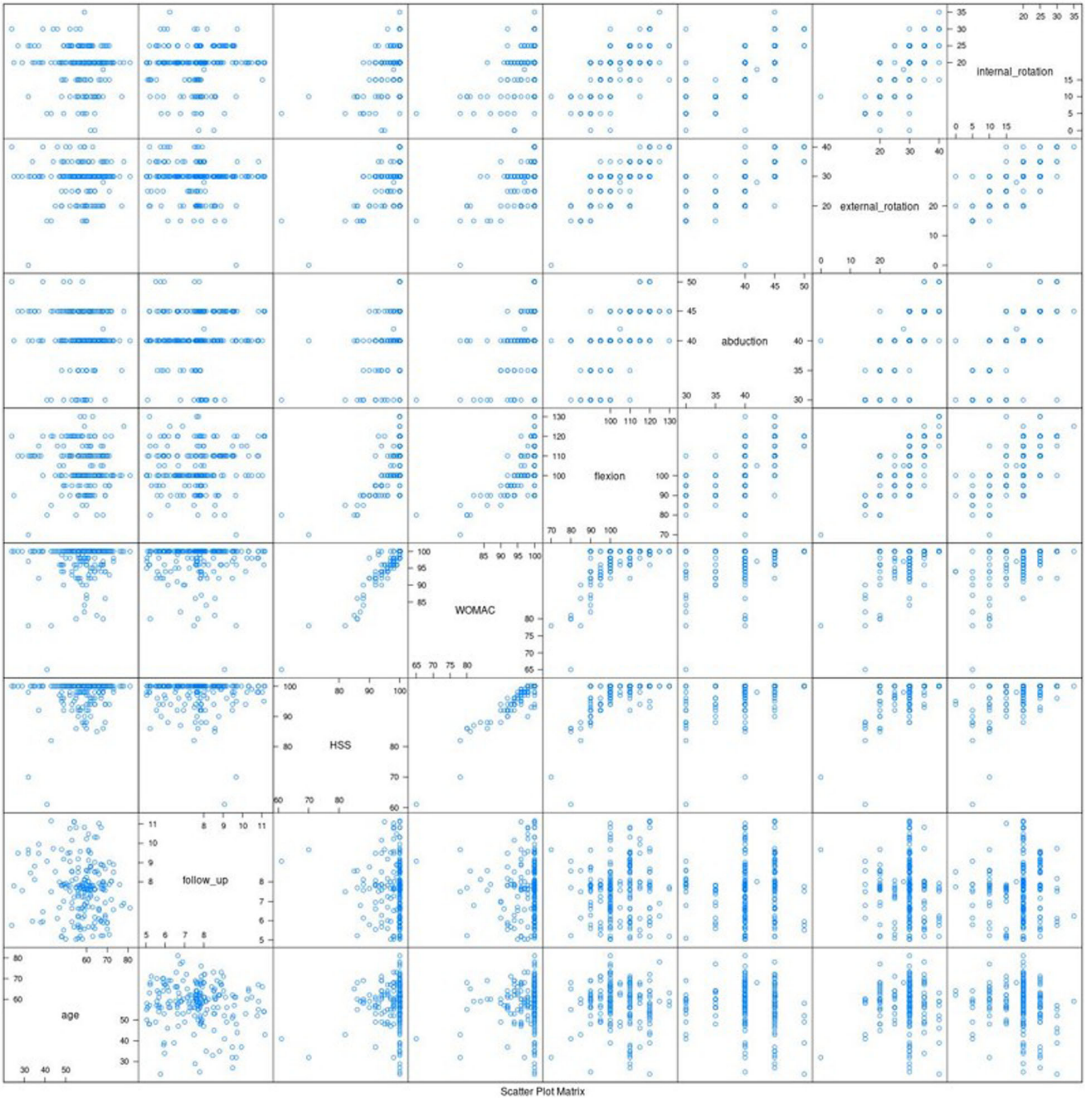
Matrix of scatter plots. The highest correlations show the values closest to the linear ascending line. The lowest correlation followed by values with random scattering of values

**Fig. 5:**
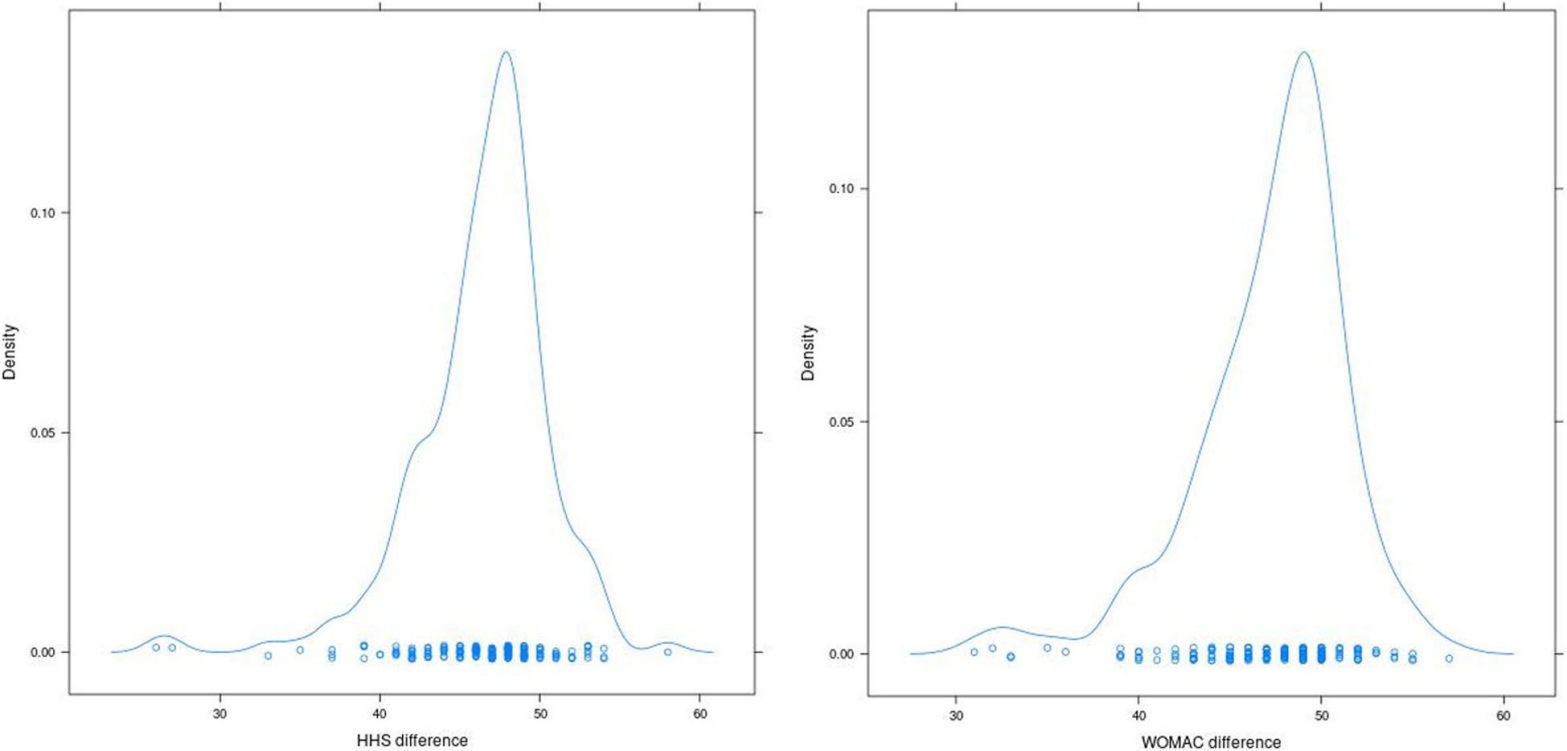
The density of HHS difference (left side) and the density of WOMAC difference (right side)

In our cohort of 198 THAs, only one revision was performed for a periprosthetic fracture. We documented an overall 99.49% Kaplan-Meier survival with an average follow-up of 7.7 years with the FIT (LimaCorporate) stem revision and any component revision as the endpoint. With the Delta PF (LimaCorporate) cup revision as the endpoint, the survival was 100%. The statistical values for range of movement, HSS and WOMAC are shown in a table (Table 2). Correlations between variables were evaluated using a scatter matrix graph (Fig. 4). There is a significant correlation between the both of clinical evaluation (HHS and WOMAC (Fig. 5) (Pearson correlation coefficient (Pcc) 0,94, Spearman correlation coefficiant (Scc) 0,91)) as well as between clinical evaluation and range of motion. The highest correlation was observed between HHS and flexion (Pcc 0,6, Scc 0,55) and between WOMAC and flexion (Pcc 0,62, Scc 0,55) and also between internal rotation and abduction (Pcc 0,7, Scc 0,7).

## Discussion

Cementless THA are currently at the forefront of orthopaedic surgery. When examining publised studies, we have not found a clinical or radiographical assessment of this implant. Haverkamp et al [6] evaluated 222 patients with an implanted Delta PF (LimaCorporate) cup, with a follow-up period of 3-21 months. They showed a cumulative survivorship of 99.5% after 1 year. In the same study, they evaluated 250 Selexys cups (Mathys) with the same follow-up time and cumulative survivorship of 87.4% after 1 year with a 7.4% revision rate for aseptic loosening [6]. A fairly large rate of aseptic loosening of the Selexys cup (Mathys) was also shown by Ilchman et al [7], where they evaluated 115 THAs with a follow-up of 2-20 months.

In their study, Benazzo et al [8] evaluated the survival of the Modulus stem with a variable neck system (LimaCorporate). They found a 98.28% survivorship of the stem with an average HHS of 96.6 in 222 patients with an average follow-up of 5 years [8]. The increased role of anatomical and short stems is becoming more and more discussed. The results of a clinical evaluation of the Fitmore (Zimmer) stem showed a HHS of 99.0 with a mean follow-up of 2 years and 3 revisions in the same period of time [9]. The evaluation of the PPF (proximal press-fit) (Biomet) stem in a cohort of 142 patients was published in 2016 [10]. Iori and Vigano showed a survivorship of 99.1% after 10 years. The PPF (Biomet) stem is a Zweymüller type of stem that has a long tradition and excellent results. There are many published studies evaluating the Alloclassic (Zimmer) stem. Both Zweymüller [11] and Delaunay [12] showed 98% survival rates of the stem in their published studies. Similarly, studies published by Suckel and Grübl [13, 14] proved 98% survival rates after 15 years. Likewise, other studies with a 9-11 year follow-up showed similar results (94% - 97%) when combined with the CSF cup (Zimmer) [15–18]. In their cohort of 154 patients with an implanted SL-plus stem (Smith & Nephew), Studers et al found a 93.5% survival rate with a mean HHS score of 92.7 and a 93.8 WOMAC [19].

In their group of 154 patients, Studers et al show a 93.5% survival of the SL-plus stem after 10 years with a mean HHS of 92.7 and a WOMAC score of 93.8 [19]. The SL-plus/Bicon-plus system (Smith & Nephew) was also evaluated by Kovoresis et al, where they published a survival of 97% after 11 years [20]. The Corail stem (DePuy) shows very good results. A 96.8% survival after 20.9 years in a cohort of 127 patients was shown by Vidalain [21]. Wangen et al published a survival of 96.9% of the Corail stem after 11 years with a mean HHS of 94 and a mean WOMAC score of 89 [22].

The amount of heterotopic ossifications occurring in our cohort might seem relatively large, however it is in concurrence with a number of previously published studies. When evaluating the Bicon/alloclassic SL system, Ottink et al showed a 71% occurrence of heterotopic ossifications (grade I. 41%, grade II. 20%, grade III. 9%, grade IV. 1%) [23]. Won Sik Choy documented an 8.7% occurrence of heterotopic ossifications with the Alumina ceramic head and Delta ceramic liner pairing [24]. In their study, White et al showed a 14.3% occurrence of ossifications (grade I. 5.5%, grade II. 6.8%, grade III. 1.7%, grade IV. 0%) [25]. White also mentioned that the risk factors for the occurrence of heterotopic ossification are the male gender and hypertrophic arthritis [25]. Kantak et al compared the influence of inter-operative lavage on the occurrence of heterotopic ossifications. In a control group (surgical wound lavage of less than 1 litre), the incidence of heterotopic ossifications was 73.3% (66 out of 90 patients). Six of those patients had grade III or IV ossifications (6.7%). In a group where a lavage of the surgical wound was performed with more than 3 litres of physiologic solution, the incidence of heterotopic ossifications was 41.5% (35 out of 85 patients), while no grade III or IV ossifications were observed [26].

## Conclusion

Evaluation of the Delta-PF-FIT (LimaCorporate, Italy) THA system with the use of ceramic-on-ceramic BIOLOX®Delta articulation surfaces shows very good outcomes. Clinical and radiographical evaluation confirms a marked subjective improvement in patient quality of life with a minimal number of complications.

## Data Availability

All data and materials is available in xls format in case of interest (coressponding author – PF). The dataset supporting the conclusions of this article is available in xls format in case of interest it is possible send by email. The datasets are not available online.

## Abbreviations

THA: Total hip arthroplasty
HHS: Harris hip score
WOMAC: Western Ontario and McMaster Universities Arthritis index
SD: Standard deviation
